# Development and validation of an in vitro model for measurements of cervical root dentine permeability

**DOI:** 10.1007/s00784-014-1194-5

**Published:** 2014-02-07

**Authors:** Holger Jungbluth, Thomas Attin, Wolfgang Buchalla

**Affiliations:** 1 Department of Preventive Dentistry, Periodontology, and Cariology, University of Zürich Center for Dental Medicine, Plattenstrasse 11, 8032 Zürich, Switzerland; 2 Department for Conservative Dentistry and Periodontology, University Medical Center Regensburg, Regensburg, Germany

**Keywords:** Dentine hypersensitivity, DH, Perfusion, Tubules, Dentinal fluid, Fluoride, Protein

## Abstract

**Objectives:**

The aim of this series of studies was the development and validation of a new model for evaluation of dentinal hypersensitivity (DH) therapies.

**Materials and methods:**

Roots from extracted human teeth were sealed with a flowable composite. In the cervical area, a 3-mm-wide circular window was ground through the seal 1 mm deep into dentine. The pulp lumen was connected to a reservoir of artificial dentinal fluid (ADF) containing protein, mineral salts and methylene blue. At increased pulpal pressure, the ADF released through the said window was collected in containers each with 20 ml of physiologic saline for a consecutive series of 30-min intervals and ADF concentration (absorption) was determined photometrically. The model was verified by three experiments. In experiment 1, the lower limit of quantification (LLoQ, coefficient of variation = 20 % and difference of 5 standard deviations (SD) from blank) of ADF in physiologic saline was determined by measuring the absorption of 15 dilutions of ADF in physiologic saline (containing 0.625 ng to 12.5 μg methylene blue/ml) photometrically for ten times. In experiment 2, long-term linearity of ADF perfusion/outflow was investigated using 11 specimens. The ADF released through the window was collected in the said containers separately for each consecutive interval of 30 min for up to 240 min. Absorption was determined and analysed by linear regression over time. In experiment 3, perfusion before (2×) and after single treatment according to the following three groups was measured: BisGMA-based sealant (Seal&Protect®), an acidic fluoride solution (elmex fluid®) and control (no treatment).

**Results:**

In experiment 1, the LLoQ was 0.005 μg methylene blue/ml. In experiment 2, permeability was different within the specimens and decreased highly linearly with time, allowing the prediction of future values. In experiment 3, Seal&Protect® completely occluded dentinal tubules. elmex fluid® increased tubular permeability by about 30 % compared to control.

**Conclusions:**

A model comprising the use of artificial dentinal fluid was developed and validated allowing screening of therapeutic agents for the treatment of DH through reliable measurement of permeability of cervical root dentine.

**Clinical relevance:**

The described in vitro model allows evaluation of potential agents for the treatment of DH at the clinically relevant cervical region of human teeth.

## Introduction

Dentine hypersensitivity (DH) is a widespread phenomenon. Its prevalence is estimated to be between 8 and 35 %. Possible reasons for this wide range are differences in populations that were studied and differences in methodology to evaluate dentine hypersensitivity, e.g. questionnaire or clinical examination [1]. DH is defined as a “short and sharp pain arising from exposed dentine in response to stimuli typically thermal, evaporative, tactile, osmotic or chemical, which cannot be ascribed to any other form of dental defect or disease” [2–5].

Its most widely accepted cause is described by the hydrodynamic theory [6,7]. According to this theory, pain-provoking stimuli are transmitted to pulpal nociceptors by rapid movements of dentinal fluid across the dentinal tubules. Most commonly, teeth showing DH have dentinal tubules open to the oral cavity [8,9].

In the cervical region in particular, open dentinal tubules are described to be caused by gingival recession [10], loss of cementum and enamel loss, as a consequence of surgical and non-surgical treatment of periodontal disease [11], and, as frequently reported and probably one of the most important factors today, by dietary acid erosion in combination with tooth brushing [12–14].

Therapeutic concepts against DH are discussed controversially, although attempts have been made to establish certain guidelines for treatment. Unfortunately, no generally accepted strategy is presently available and dentists’ and dental hygienists’ knowledge of pathogenesis and treatment options for DH is limited [2]. Although dentists treat cases with hypersensitive dentine areas using different invasive treatment regimens, a systematic and evidence-based treatment concept is still lacking.

Another problem in the field of DH is the lack of strong evidence for treatment efficacy of the proposed treatment regimens. Some well-designed clinical studies have shown desensitizing dentifrices to have a beneficial effect, if used correctly in combination with removal of predisposing factors [15–17]. There is no generally accepted index for pain elicitation and assessment. This fact presents a limitation of clinical investigations [18].

In addition to clinical studies, in vitro studies have their eligibility in testing different treatment protocols before clinical trials are performed. Operational sequences can be standardized more easily in vitro than in a clinical setting. In vitro studies on this subject usually are designed to measure dentinal perfusion and the sealing of patent dentinal tubules. The most common model used is a hydraulic conductance measurement approach or modifications thereof [19]. In this model, the fluid shift or the perfusion across human mid coronal dentine is measured by movement of an air bubble in a micropipette that is linked between a pressurized container and a fluid reservoir on one side and the specimen on the other side.

The frequency and diameter of dentinal tubules have a strong influence on the permeability, but its variability is high according to the location in the tooth. Regions next to the pulp space of the coronal part of dentine show the highest density of tubules per square millimetre, while on root dentine, the density is lower and decreases further from cervical to apical [20,21]. In most cases, coronal rather than cervical dentine was used in combination with the hydraulic conductance model. In the conventional hydraulic conductance model, a possible leakage through the connection of specimen holder and specimen is difficult to detect because it may not be visible. Thus, the accuracy and reliability of reading the movement of the air bubble might be limited in these cases. A visual control of such leakage would be preferable.

While some investigators worked with a protein component in dentine perfusion assays and also showed that their topical use on permeable dentine reduces its hydraulic conductance [22,23], a dentine perfusion model using a dentinal fluid-like medium is not described to date. In order to facilitate different precipitation mechanisms in dentinal tubules [9,24] during effectiveness tests of agents resolving DH, a liquid medium similar to dentinal fluid has some advantages in a dentine perfusion assay.

The aim of the present study was the development of an improved dentinal perfusion model. The perfusion should be carried out on human cervical root dentine with a perfused solution similar to human dentinal fluid (artificial dentinal fluid). Also, a possible undesired leakage through the connection of specimen holder and specimen should be able to be identified.

## Materials and methods

### Description of the in vitro testing method and apparatus

#### Specimen preparation

Forty-two caries-free upper and lower third molars were extracted and stored in 0.1 % thymol solution at 4 °C until use. The crowns of the teeth were removed at a level 1 mm above the cemento-enamel junction with a low-speed diamond saw (Isomet, Bühler, Germany). The resulting roots were used for the experiment. During specimen preparation, the roots were kept moist except for the short periods of using dental adhesive or cyanoacrylate glue. The pulp tissue of the roots was removed with barbed broaches and #15 Hedström files, and the roots were rinsed with physiologic saline.

**Fig. 1:**
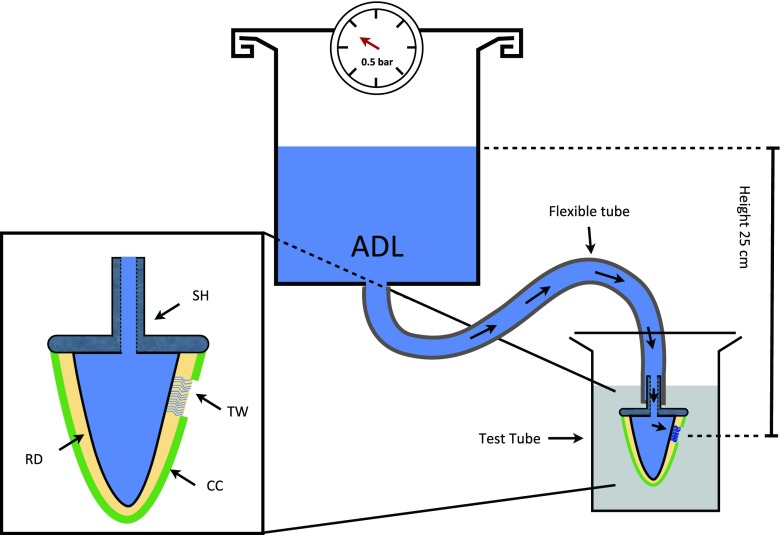
Experimental set-up. The pressurized ADF reservoir is connected to the specimen via a flexible tube. The specimen is submerged in a test tube filled with 20 ml physiologic saline. The height between the upper level of ADF and specimen is adjusted to 25 cm. The close-up view of an enlarged cross section through the specimen is shown in detail: specimen holder (*SH*), composite coating of the root (*CC*), test window (*TW*) and root dentine (*RD*)

The roots were dried with gentle air stream, and a fine layer of cyanoacrylate glue was applied on the cut surface of the root with the tip of a dental probe. The roots were then glued on custom-made scanning electron microscope specimen holders, which had a concentric hole through their shafts. Thus, drainage of the pulpal space through the shaft of the specimen holder was possible (Fig. 1). The junction between the root and the specimen holder was additionally reinforced with methacrylate resin, extending 1 mm onto the root surface.

After intermediate storage in tap water, remnants of the periodontal ligament and the cementum of the roots were removed with a sharp hand scaler. The root surfaces were then brushed thoroughly with a nylon brush and slurry of pumice and tap water. After rinsing the root surfaces with tap water, they were etched with 37 % phosphoric acid for 1 min and then again rinsed with tap water for at least 1 min. A dental adhesive (Syntac Classic, Ivoclar Vivadent AG, Schaan, Liechtenstein) was applied according to the manufacturer’s instructions. The whole root surface was then coated with a layer of approximately 1 mm thickness using a white-coloured flowable resin composite (Filtek Supreme Ultra Flow, 3 M ESPE AG, Seefeld, Germany) and light-cured in order to completely seal dentine tubules and the openings of the apical foramina.

Subsequently, a circular window of about 3 mm in diameter was ground just below the cemento-enamel junction through the composite layer and 1 mm deep into the underlying root dentine using a cylindrical diamond burr (ISO no. 314 110 544 015, Intensiv SA, Lugano, Switzerland). The depth of the window within the dentine was measured with a 1-mm branded periodontal probe, taking the border between the white composite and the yellow-coloured root dentine as the reference.

The specimens were then stored in physiologic saline until use. The experiments started a maximum of 1 week after specimen preparation. Just before use, the pulp space and the exposed dentine surface of the circular window were rinsed for 3 min with 17 % EDTA (Kantonsapotheke, Zurich, Switzerland). In order to rinse the pulp space, this was done with a 10-ml syringe and a blunt irrigation cannula (Endo-EZE, Ultradent Products Inc., South Jordan, UT, USA) that was slowly entered into the pulp space. During every application of solution into the pulp space, care was taken that no air bubbles were carried into the specimens’ pulp spaces. After the rinse with EDTA, the pulp spaces and the test windows were thoroughly rinsed with at least 10 ml deionized water for 3 min.

#### Composition of the artificial dentinal fluid

ADF was developed in a preliminary study and showed chemical stability over 3 months without precipitation. The solution contained 2.5 g/l methylene blue, 35 g/l albumin (bovine serum albumin, Fraction V, Art. 1.12018, Merck KG, Darmstadt, Germany), 2.5 mM PO_4_^3−^, 2.35 mM Ca^2+^, 4.7 mM Cl^−^, 1.0 mM K^+^ and 3.81 mM Na^+^. The pH of the ADF was 7.0 and tested with a standard pH electrode (713 pH Meter, Metrohm, Zofingen, Switzerland). To avoid irreversible precipitation of some of the ingredients, albumin, inorganic compounds and methylene blue were separately solved in deionized water prior to mixing. The osmolarity of the solution was 32 mOsm (Fiske one-ten, Fiske Associates, Needham Heights, MA, USA).

### Testing method and apparatus

The pulp space of each specimen was filled with ADF and its nozzle was connected with a pressurized ADF reservoir by a flexible tube (inner diameter 2 mm, no. 1337, Novoplast Schlauchtechnik GmbH, Halberstadt, Germany). This system was then completely filled with ADF (Fig. 1).

The pressurized ADF reservoir was positioned 25 cm above the specimen, resulting in a net pressure of 25 hPa within the specimen. The reservoir was connected to a compressed air system via a pressure-reducing regulator (Type 10, Bellofram Corporation, Burlington, MA, USA), allowing increase and control of the pressure in the system.

Thereafter, the specimen was put into a centrifuge tube containing a given amount of physiological saline (20 ml). Depending on the individual experiment, the pressure within the system was increased to a higher level and, thus, the ADF was perfused through the dentine at the test window. The resulting dye concentration of the saline solution in the centrifuge tube was measured with a spectrophotometer and taken as a measure for the ADF permeability of the cervical root dentine.

#### Experiment 1: validation of the spectrophotometric assay

Dyeing of saline solution in the centrifuge tube was assessed by measurement of the photometric absorption with a spectrophotometer at a wavelength of 664 nm. In order to validate the reliability of the spectrophotometric assay, the lower limit of quantification (LLoQ) was determined. The LLoQ describes the lowest concentration of a substance a method can confidently quantify. In this study, the LLoQ was defined as the lowest concentration of ADF with acceptable repeatability given by an intra-assay variation (coefficient of variation, CV%) of <20 % and, at the same time, could be clearly differentiated from the blank value by 5 standard deviations (=mean of blank + 5 × SD) [25].

To determine the LLoQ, a series of 15 dilutions of the ADF was mixed, comprising methylene blue concentrations from 0.625 ng/ml to 12.5 μg/ml. The dilution medium was physiologic saline, which also served as the blank. The absorption of the 15 dilutions was measured successively in the direction of ascending methylene blue content using a standard photometer (U-2010, Hitachi, Ltd., Tokyo, Japan). The measurements were repeated ten times. With the resulting absorption values, the mean and standard deviation (SD) of the measurement series were calculated for each of the 15 dilutions. The coefficient of variation was calculated in percent by dividing the standard deviation by the mean. In order to calculate the 20 % threshold of CV%, a least-square-based best fit equation was calculated using an online curve fitting software (zunzun.com).

#### Experiment 2: perfusion variation in time: determination of perfusion drift

**Fig. 2:**
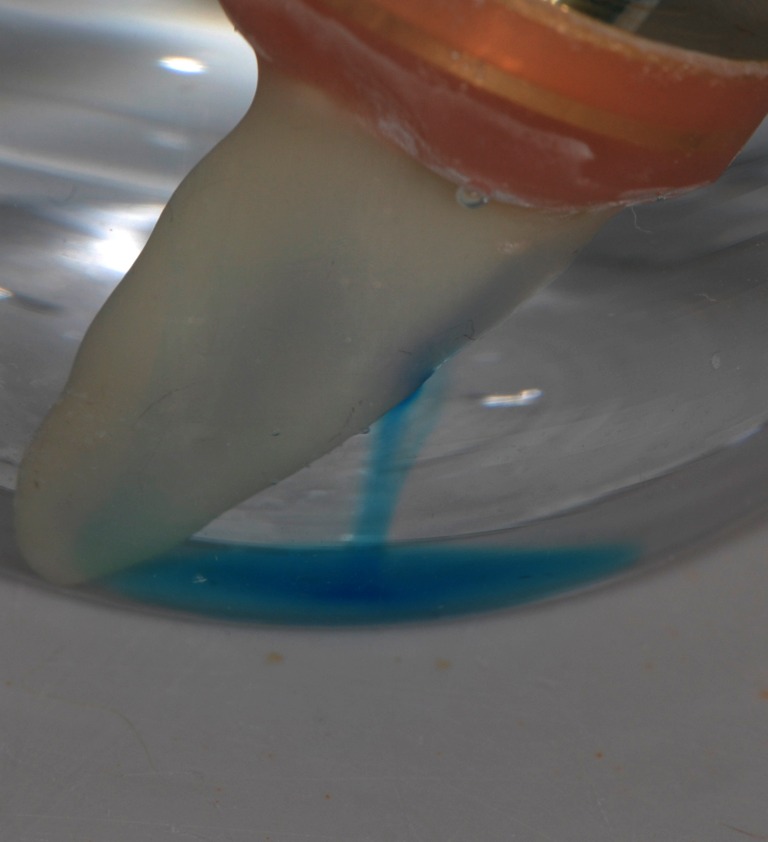
Specimen during perfusion. After some minutes of perfusion, the outflow of the stained ADF becomes visible, sinking slowly to the floor of the test tube

**Fig. 3:**
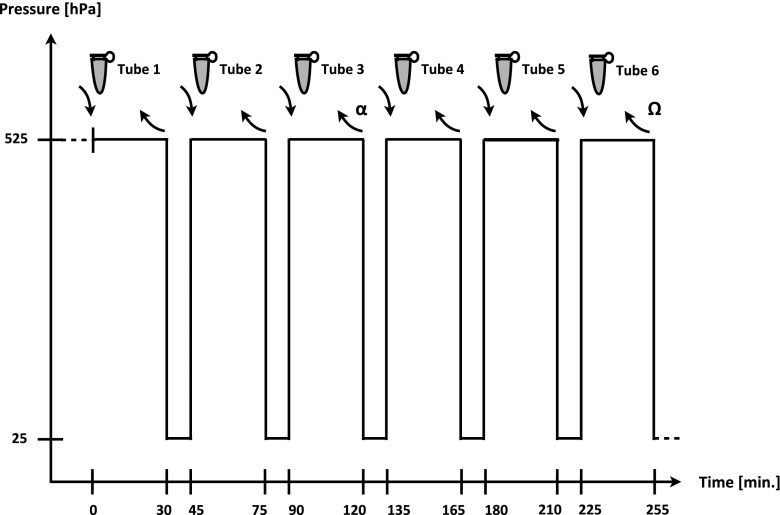
Cycling of the pressure within the specimen with time. The test begins at high pressure (525 hPa). When a continuous flow of ADF is observed, the specimen is placed into tube 1 and time measurement is started (time point 0 min). In each tube, the specimen rests for 30 min at high pressure, followed by a 15-min low-pressure interval in physiologic saline as an intermediate storage medium in another tube (not shown). End point of experiment 3 is signed by alpha (*α*) and of experiment 2 by omega (*Ω*)

In the second experiment, the stability and drift of the perfusion through the dentine was assessed over a longer time period. A pressure of about 525 hPa was established in the pulpal space of 11 single specimens (*n* = 11) that were seated in a storage vessel filled with physiologic saline by increasing the pressure in the ADF reservoir to 500 hPa. When a continuous stream of ADF could be seen passing through the specimen window, the experiment was started (Figs. 2 and 3). Hitherto, the specimens were rinsed with physiologic saline from outside, gently dried and immediately put into a new centrifuge tube (tube 1) with 20 ml of fresh physiologic saline, and the time was measured beginning with this starting point (time = 0 min in Fig. 3). The specimens were kept in the tube for 30 min and then removed (at time point 30 min in Fig. 3) and stored transiently at reduced, environmental pressure conditions (=25 hPa of the apparatus) in a storage vessel also containing fresh physiologic saline. After 15 min of storage, the specimens were put into a new centrifuge tube (tube 2, at time point 45 min) with 20 ml fresh saline and the pressure was raised again to 525 hPa. After another 30 min (at time point 75 min), the specimens were removed from tube 2. These alternating pressure conditions were repeated five times (up to time point 255 min), and the absorption caused by the dye stained ADF in each centrifuge tube (tubes 1 to 6) was measured with the photometer at a wavelength of 664 nm. During perfusion time, the absence of ADF perfusion beyond the previously described window was determined visually. In case of any leakage beyond this window, e.g. through a gap between specimen holder and root, the respective specimen was discarded.

The resulting values of the measured absorption were characterized with “EXT” (extinction) and the according time point of measurement, e.g. absorption at time point 30 min = EXT(30). Data of the absorption measurements were statistically analysed with the software PASW Statistics version 18.0 (IBM Corporation, Armonk, NY, USA). A linear regression analysis was calculated for each specimen from EXT(30) to EXT(255) for a possible decrease of perfusion through the dentine over time. The coefficient of determination (*R* ^2^) was calculated in order to obtain information about the linearity of the observed perfusion decrease.

#### Experiment 3: test of tubular occlusion

The third part of the experiment was based on the findings of experiment 2 (perfusion variation in time). It was carried out like experiment 2, but with two treatment groups containing 11 specimens each. In contrast to the former experiment, the dentine at the test window was treated with two different agents after the second high-pressure interval of the test and within the following 15 min of low-pressure interval (within time point 75 min and time point 90 min in Fig. 3). In groups 1 and 2, elmex fluid® (acidic fluoride solution containing 10,000 ppm F^−^; GABA International AG, Therwil, Switzerland) and Seal&Protect® (polymer-based light-curing sealing material for exposed dentine of hypersensitive teeth; DENTSPLY DeTrey GmbH, Konstanz, Germany) were used, respectively. In the first group, teeth were gently dried using a soft air stream from a three-way air/water syringe for 5 s; then, a full drop of elmex fluid® was applied on the test window with a microbrush applicator (Disposable Applicators, 3 M ESPE AG, Seefeld, Germany) and left for 60 s. Subsequently, the fluid was removed carefully with dry microbrush applicators and the specimen was put back into the storage medium (physiologic saline). The teeth in the second group were dried in the same way with a soft air stream, and Seal&Protect® was applied and light cured twice according to the manufacturer’s instructions. After that, each specimen was again stored in the storage vessel until the 15 min of this interval of low pressure was completed (time point 90 min in Fig. 3). Subsequently, the specimen was placed in a new tube (tube 3), and a last high-pressure interval was carried out (end point = time point 120 min). Absorption of the solutions in tube 1 to tube 3 was again measured with the aforementioned photometer. Experiment 3 was done by the same operator and under the same conditions like those in experiment 2, and both experiments were conducted in direct chronology.

**Fig. 4:**
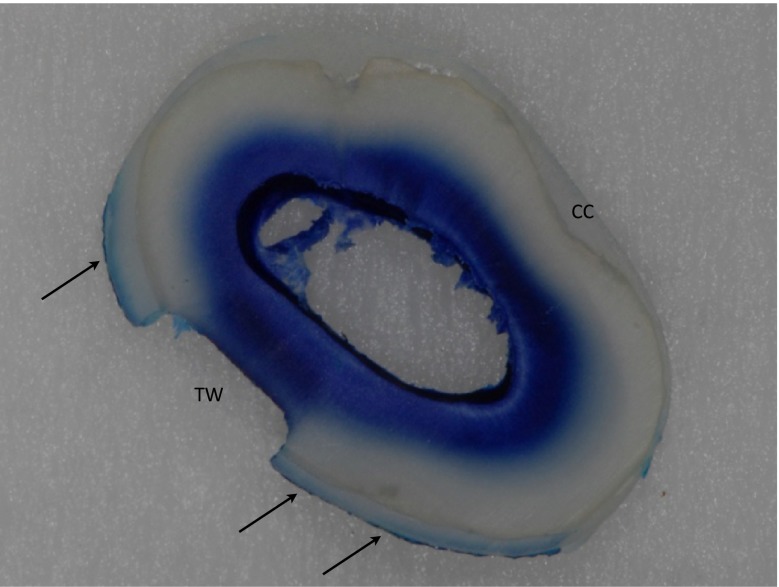
Cross section at the level of the test window (*TW*) of one specimen after use. No leakage has occurred between root surface and composite coating (*CC*). Blue-dyed ADF has penetrated the whole length of dentinal tubules at the test window. In other dentine areas, penetration is less deep, to about half the distance from the pulp space to the outer root surface. Minor adsorption of blue dye to the composite coating around the test window is visible (*arrows*)

All specimens were sectioned under water cooling with a low-speed saw (IsoMet, Bühler GmbH, Düsseldorf, Germany) perpendicular to the long axis directly after the experiment. The resulting slides were inspected using a stereomicroscope (Stemi 1000, Zeiss, Oberkochen, Germany) to exclude perfusion of the ADF beyond the test window or between the tooth and composite coating (Fig. 4).

For statistical analysis in this part of the experiment, data from experiment 2 of the first three tubes (tubes 1–3) served as the negative control (no treatment). This was legitimate, because here, no treatment was done on the test window between time point 75 min and time point 90 min. Data were again transferred to the software PASW Statistics version 18.0, and non-parametric statistical tests were used.

Within the three resulting groups (two test groups and one control group), for each specimen, an expected value EXTexp(120) was calculated from the EXT(30) and the EXT(75) values. This was done according to the following equation:

$$ \mathrm{EXTexp}(120)=\mathrm{EXT}(75)-\left[\mathrm{EXT}(30)-\mathrm{EXT}(75)\right] $$

The resulting EXTexp(120) data sets were compared with the effectively measured EXT(120) data sets pertaining to each group (Mann-Whitney *U* test, *p* = 0.05). Analysing the control group, this comparison allowed verification of the accuracy of the expected values [EXTexp(120)]. Within the test groups, this comparison revealed the treatment effect. Differences of expected and effectively measured values for EXT(120) were then calculated in percentage of the expected values [EXTexp(120)] and outlined to a box plot.

Furthermore, a Mann-Whitney *U* test was calculated between the control group and each of the two test groups of these percent values. Significance level was set at *p* = 0.05.

## Results

For the study protocol, 42 specimens were collected. Four specimens could not be used, because no perfusion was visible through the test window and thus they were discarded. Another five specimens were discarded due to leakage of ADF through the gap between the specimen holder and root. The remaining 33 specimens showed regular perfusion of ADF. Cross sections cut through all specimens after the perfusion test proved that no leakage outside the desired pathway had occurred in the included specimens.

### Experiment 1: validation of the spectrophotometric assay

**Fig. 5:**
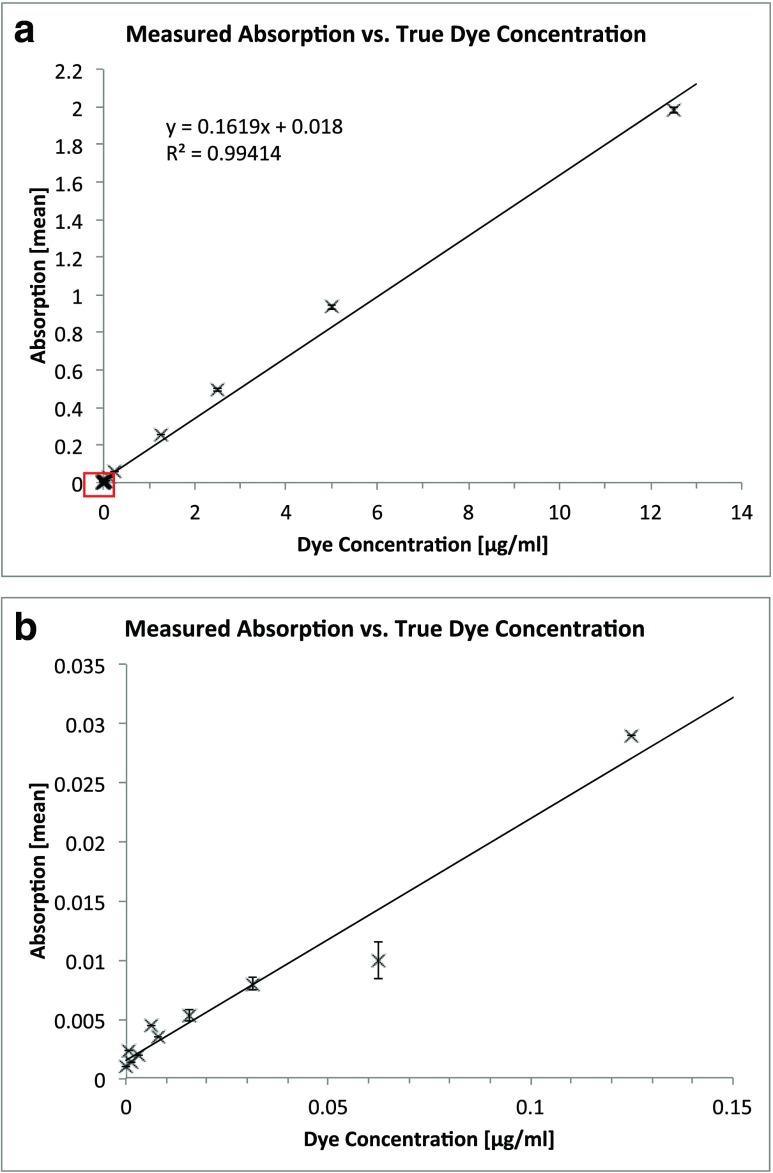
**a** Absorption means plotted against the true methylene blue concentration. The standard deviations (whiskers) are too small to be visible in this diagram. The absorption is linearly related to the concentration of methylene blue (*R* ^2^ = 0.994). *Red square* highlights the data shown in Fig. 5b. **b** Detail from Fig. 5a. Only the lowest ten values are shown. *Whiskers* indicate standard deviations of the absorption means

Measured absorption versus true concentration of the methylene blue dye from ADF diluted in physiologic saline (Fig. 5a) shows a linear relationship along all concentrations with *R* ^2^ = 0.994. In this diagram, the standard deviation of every mean was too small to be visible and the smaller values overlap (red square). Therefore, Fig. 5b shows the smaller values in Fig. 5a in greater detail. In Fig. 5b, the standard deviations of the means become visible.

**Fig. 6:**
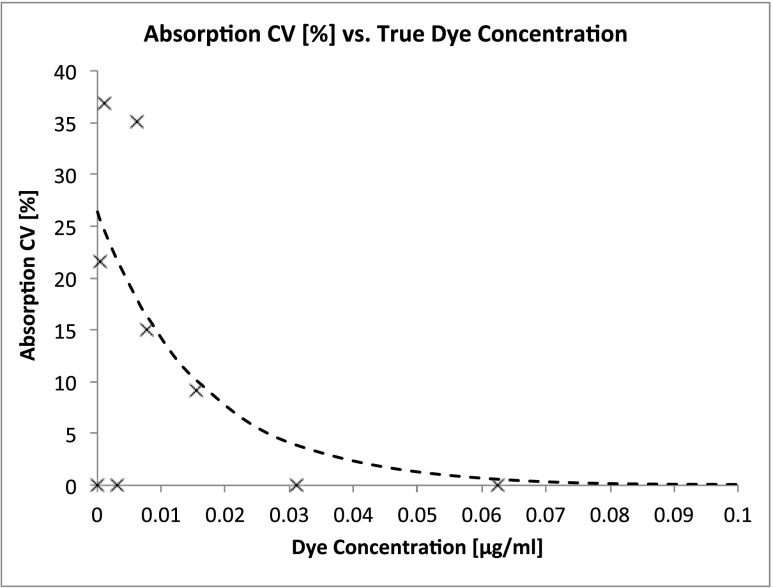
Determination of the lower limit of quantification. The coefficient of variation (CV%) of the absorption means is plotted against the true dye concentration. The diagram shows only the low dye concentrations, in order to enhance visibility. A fit CV % = 21.400 ⋅ *e* ^− 61.8423 ⋅ *x* + 0.2083^, with *x* being the dye concentration [micrograms per millilitre], *dashed line*) shows an exponential decrease with time. The CV% falls below 20 % with dye concentrations higher than 0.005 μg/ml

The coefficient of variation (CV%) of the absorption plotted against the true dye concentration (Fig. 6) shows a decreasing CV% with increasing concentration (with the exception of two absorption values). Only the lowest concentrations are shown in Fig. 6 in order to provide more detailed information close to the blank. An exponential algorithm was selected following least-square routines resulting in an equation which allowed for calculation of the 20 % threshold for the CV%. The resulting fit (CV % = 21.400 ⋅ *e* ^− 61.8123 ⋅ *x* + 0.2083^, with *x* being the dye concentration [micrograms per millilitre]) is shown by a dashed line (Fig. 6). According to the formula, the CV% falls below 20 % for dye concentrations higher than 0.005 μg/ml.

The standard deviation of the blank was <0.000. Due to the low standard deviation of the blank, absorption was above blank value + 5 × SD for all measured dye concentrations and, hence, was distinguishable from blank absorption. Therefore, the LLoQ could be determined as a dye concentration of 0.005 μg/ml, corresponding to an absorption of 0.003.

### Experiment 2: perfusion variation in time: determination of perfusion drift

**Fig. 7:**
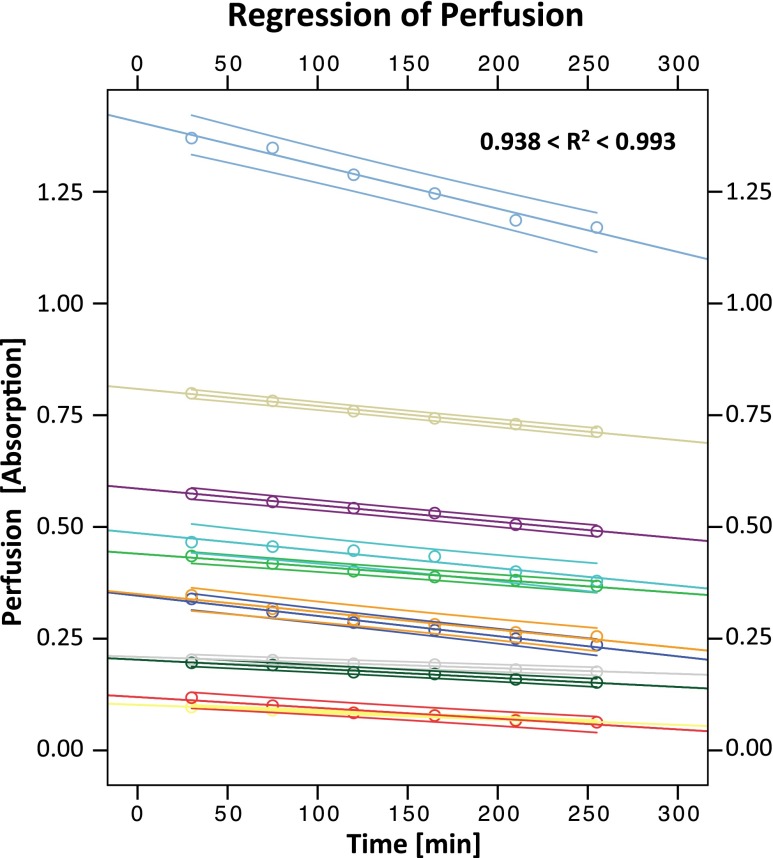
Absorption (perfusion) of individual specimens over time (*circles*) with corresponding linear regression lines and 95 % confidence intervals. For different specimens, the perfusion starts at different levels. The regression of the perfusion appears highly linear (0.938 < *R* ^2^ < 0.993) with a similar slope between −0.00013 and −0.00097 for all specimens. Please note that the values of some of the specimens are very close to each other and therefore overlap in the diagram

In experiment 2, no measurement was below the LLoQ (absorption of 0.003). The perfusion showed a slight, but continuous decrease with time (Fig. 7). The coefficient of determination (*R* ^2^) was between 0.938 and 0.993 (median = 0.981). This indicates that the decrease was highly linear. The modulus of the slope of the decrease was smaller than 0.001 for all specimens.

### Experiment 3: test of tubular occlusion

EXT(30) and EXT(75) values were between 0.02 and 1.592. EXT(120) values ranged from 0.194 to 1.631 in the elmex fluid® group and from 0.002 to 0.008 in the Seal&Protect® group (median = 0.004). The values of the Seal&Protect® group are very close or even below the LLoQ and were therefore considered to be 0. Calculated EXTexp(120) values and measured EXT(120) values of the control group were statistically not different, indicating that prediction of EXT(120) values from the means of EXT(30) and EXT(75) values is reliable. In the two test groups, significant differences between EXTexp(120) and EXT(120) were present, indicating treatment effects in both groups.

**Fig. 8:**
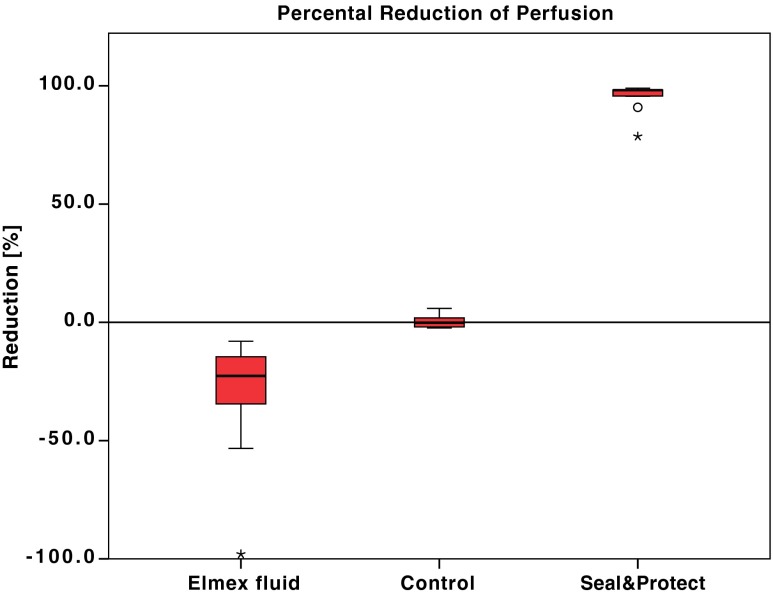
Box plot depicting the percent reduction of perfusion after 120 min [EXT(120)] compared with the calculated EXTexp(120). *Boxes* confine upper and lower quartiles. *Horizontal lines within boxes* indicate medians, and *whiskers* indicate the highest and lowest sample values, respectively, that are not assigned to be “outliers” or “extremes”. “Outliers” (indicated as *circles*) are defined to be distant between one and a half to three box lengths from either end of the box; “extremes” (indicated as *asterisks*) differ more than three box lengths from either end of the box. Values of the elmex fluid® group are negative, indicating that perfusion has increased. The control group showed no reduction of the expected value EXTexp(120). Seal&Protect® reduced perfusion by nearly 100 %

In the control group, the measured values showed a negligible difference to the expected values (−0.3 ± 2.7 %; mean ± SD, Fig. 8). In the Seal&Protect® group, perfusion was decreased (−95.6 ± 5.8 %), and in the elmex fluid® group, the perfusion was increased (29.9 ± 25.0 %). Thus, Seal&Protect® seems to (nearly) completely block dentinal tubules for ADF perfusion, while elmex fluid® enhances perfusion significantly.

## Discussion

The current study presents a new method to quantitatively measure cervical dentinal perfusion. The main long-term objective of such measurements is to obtain knowledge about the effectiveness of different treatment protocols against dentine hypersensitivity. Although the presented in vitro model cannot completely mimic the clinical situation, it serves as a possible tool to address specific questions in the field of DH, particularly because some parameters closely resemble the clinical situations, i.e. participation of cervical dentine and dentinal fluid.

The model provides the possibility to measure how well a topical therapeutic agent occludes dentinal tubules. Other published models, e.g. the hydraulic conductance model, have the same goal, but work with different parameters, resulting in some advantages and also some disadvantages. In the present model, an artificial dentinal fluid is used, because some products against DH are thought to interfere with particular components of dentinal fluid in order to be effective. These components of the ADF that are most likely involved in precipitation or other occlusion events are minerals like calcium and phosphate and serum proteins like albumin. The exact composition of the ADF was based on physiological specifications of the interstitial fluid [26], because studies on the composition and physiological outflow of human dentinal fluid have shown that dentinal fluid comprises nearly the same components as interstitial fluid [27–30].

Additionally, the ADF solution was intended to meet a second criterion. Beside the favoured property of passing through the dentinal tubules and resembling to dentinal fluid, it should be well coloured to allow photometrical analysis and at the same time visual control of perfusion and possible leakage. The dye used, methylene blue, has a very small molecular size and is well soluble in aqueous solutions. It shows a slightly acidic pH, which was buffered to a neutral level by adding phosphate buffer to the ADF solution. The mixing of ADF had to follow a specific mixing protocol, but then, ADF showed long-term stability of more than 3 months. During this time, it was stored at 4 °C in an amber glass bottle and checked weekly for possible precipitations, which did not occur.

The blue staining of the methylene blue allowed good visual control of the ADF perfusion through the dentine during the test. When perfusion was started, the dentine surface on the test window slowly became more and more blue, until a continuous flow of blue colour originating from the test window could be observed. Leakage occurred through the connection between the root and the scanning electron microscope specimen holder in five specimens. These specimens were easily identified and could therefore be discarded. In a preliminary study (data not shown), the root surfaces were not covered with the flowable composite but with nail varnish. This led to leakage of ADF in many samples through the interface between the root surface and nail varnish. Frequently, this leakage occurred from the apex to the test window. This event could successfully be avoided using a dentine bonding system in combination with the flowable composite. Cross sections of the specimens showed clearly that among all specimens included in the study, there was no leakage outside the desired pathway.

To remove a possible smear layer from the test window and from the pulp space walls in order to allow perfusion, the specimens were rinsed for 3 min with 17 % EDTA. An important issue was the complete removal of this EDTA from both sides. Therefore, the specimens were thoroughly rinsed with ultrapure water for at least 3 min. The effectiveness of removing a solution from the specimens’ pulp space was tested in a preliminary test to this study. Hitherto, the pulp spaces of three specimens were filled with a dye solution and the volume of ultrapure water needed to achieve unstained drain of the pulp space was measured. After the use of 3 ml of ultrapure water, a completely clear and unstained solution was drained from the specimens. Within the study, the pulp spaces were rinsed with 10 ml ultrapure water over a period of 3 min, thus, it was expected that EDTA was removed completely from the dentine surfaces.

Even though the smear layer was removed using 17 % EDTA, no perfusion occurred during the test in four specimens, which were discarded for this reason. After sectioning these specimens, it was observed that the perfusion of the ADF has not reached the exposed dentine surface at the test window, as it has in all other specimens (Fig. 4). The penetration of the ADF was visible in these four specimens only to about half the distance from the pulp space to the external root surface, similar to those areas that were not underlying the test window (Fig. 4). From these observations, it can be followed that no patent tubules were opened during specimen preparation. Most likely, tubular sclerosis in the exposed root dentine or incomplete removal of cementum during specimen preparation (e.g. in teeth with an individually thicker cementum layer) may be responsible. While an exposure of just cementum would have been visible in the sections but was not the cause, tubular sclerosis is the more likely reason. Even in Fig. 4, a small area at the test window showed no perfusion (unstained exposed dentine area), while the root dentine was exposed clearly and the removal of the smear layer should be as effective, as at the adjacent areas due to the specimen preparation protocol. The depth of the test window seems to be a little less compared to the adjacent areas that show perfusion, and maybe, here, between the area showing perfusion and the area without perfusion, a border in exposure depth is crossed, where non-patent tubules turn into patent tubules. Whereas this was not specifically investigated in the study, these considerations remain hypothetic and could be a subject of further investigation.

A determination of the LLoQ of the spectrophotometric assay was done in order to assess the lowest concentration of methylene blue that could reliably be measured. The LLoQ is a lab-dependent parameter, which should be determined from every investigator in his own laboratory prior to working with this model. The results of experiment 1 show that the spectrophotometric assay is well qualified for the model due to its property to measure the smallest concentrations of methylene blue reliably.

Experiment 2 of this study indicated that there is a constant and linear decrease of the perfusion through the dentinal tubules. In a preliminary study, the authors observed that the perfusion completely ceases in most specimens when a prolonged testing time, e.g. 1 or 2 days, was chosen. This effect was not yet described in the literature, but other authors already showed a significant decrease of hydraulic conductance of dentine after the single use of different plasma proteins, full plasma or serum [23]. The reason for the constant decrease of perfusion could be a possible precipitation of ingredients of the ADF within the dentinal tubules or at their walls, but to date, this remains unclear. This effect leads to a limitation of the present model regarding the applicability on treatment regimens that need a longer period of residence time. Treatment protocols or agents that are applied within 15 or up to e.g. 30 min can be adequately tested with the present model or modifications thereof. Treatment protocols exceeding this treatment or application time to a multiple (for example, the daily use of a desensitizing dentifrice) cannot be tested, because the decrease of the perfusion is no longer reliably predictable over longer time periods, e.g. days or weeks.

Another presumed limitation of the method is the high pressure that is used for the perfusion of ADF through the dentinal tubules combined with an outward directed fluid flow. Such an increased perfusion pressure is not within physiologic conditions. Possibly, microtags or precipitates that could loosely occlude the entrance of dentinal tubules and clinically already provide a relief of DH might be underestimated in the presented model and thus reveal no prevention or reduction of perfusion. In clinical situations, the stimuli responsible for DH result in a dentinal fluid movement or flow across dentinal tubules that can be directed either inwards or outwards. The former situation can furthermore not be simulated with the present model. The solely outward directed flow combined with the high pressure could thus result in underestimation of the efficacy of desensitising agents, when tested with the present model.

For application of the model, a pressure difference between pulp space and test tube is inevitable. It would certainly be possible to reduce the maximum pressure to some degree. Experiment 1 revealed that the photometric measurement is very sensitive, and therefore, less staining of the saline would still lead to reliable results. In this context, a reduction of the volume of saline in the test tube could be employed, in order to cause sufficient staining of this solution. The absorption baseline values EXT(30) were in average over 100-fold higher than those of the LLoQ. Therefore, the parameters of the current study set-up could still be adjusted to favoured levels. An advantage of the high pressure in this model is, on the other hand, that if a product or treatment regimen shows a tight occlusion of the dentinal tubules in the test model, it is likely that also in a clinical situation, this kind of treatment would be effective against DH.

The results of experiments 2 and 3 lead furthermore to the assumption that the method presented is valid within its aforementioned limitations. The calculation of the expected staining of tube 3 [EXTexp(120)], based on the absorption values EXT(30) and EXT(75), proved highly accurate. The difference between the expected (calculated) perfusion at 120 min [EXTexp(120)] and the measured perfusion [EXT(120)] was only −0.3 ± 2.7 % within the control group. Seal&Protect® has shown to be an appropriate positive control. The 1-min action of elmex fluid® on the dentinal surface showed no reduction, but a significant increase of dentinal perfusion. This is an important finding and can be explained by the low pH (pH 3.9) of elmex fluid®. The concentrated solution seems to demineralize dentine and thus slightly open dentinal tubules. Seal&Protect® on the other side seems to effectively occlude dentinal tubules. The absorption measurements in the Seal&Protect® group gave values from 0.002 to 0.008 (median = 0.004), while the LLoQ was determined to be 0.003. Subsequently, it was hypothesized that the tubules were totally sealed in these specimens. Nevertheless, a very small absorption could be measured in this group. The cause for this finding might be based on an observation that was made during the test. During the first two perfusion intervals (time point 0 min to time point 30 min and time point 45 min to time point 75 min in Fig. 3), the specimens were immersed in a solution, which continuously accumulated dye. In this period, the outer surface of the specimens, i.e. the composite layer, adsorbed the blue dye to a very small extent. Each time the specimens were removed from one tube and inserted into the next tube, they were thoroughly rinsed with saline and gently dried with a cellulose towel to exclude possible dye carry over from one tube to the next. Although a carry over from the liquid phase was excluded with this procedure, the dye adsorbed at the outer composite surface of the specimens may have been released into the next saline container and therefore be measured by the spectrophotometer. However, the composite surfaces were not polished. It is likely that polishing of the specimen surfaces would reduce this carry over effect.

For statistical analysis of experiment 3 of the present study, data from experiment 2 of the first three tubes served as the negative control (no treatment). This was considered as legitimate, because no treatment at the test window occurred between time point 75 min and time point 90 min. Another approach would have been to test a separate group within experiment 3 with another 11 specimens as negative control without treatment. The authors abstained from this, because it can be assumed that no differences between such a separate group and data from experiment 2 are to be expected. In future studies with the present method or modifications thereof, a separate negative control group is indispensable, not least because presumably no data corresponding to those of experiment 2 in the present study will be available.

An advantage of the current method is certainly the high detectability of each kind of perfusion and leakage due to the content of methylene blue within the ADF. Thirty-three specimens were included in the study, while five specimens had to be excluded and be replaced due to leakage through the connection between the root and SEM specimen holder. The roots were glued on the specimen holder with cyanoacrylate, as it is mostly done in studies working with the hydraulic conductance model. The connection was additionally coated with methacrylate resin. Nonetheless, leakage occurred in five specimens at this connection, which could only be detected due to the staining of the ADF. If the solution had been clear, as for example saline or buffered saline, these five specimens would have led to faulty measurements. Studies working with the hydraulic conductance model regularly use unstained and clear solutions, which may lead to unnoticed leakage.

## Conclusions

An in vitro model allowing measurements of cervical dentinal perfusability using a new artificial dentinal fluid as a perfusion medium containing a blue dye was presented. The model tested proved valid and, therefore, may be suitable in order to examine treatment regimes against DH which are based on tubular occlusion.
